# Shen fu injection for patients with septic shock: Protocol for an updated systematic review

**DOI:** 10.1097/MD.0000000000017004

**Published:** 2019-09-20

**Authors:** Xiaojun Wang, Canfeng He, Yinhe Cai, Dazhong Sun, Guangyun Hu, Jian Zhou

**Affiliations:** a The First Affiliated Hospital of Guangzhou University of Chinese Medicine; b Guangzhou University of Chinese Medicine; c The Second Hospital of Traditional Chinese Medicine in Guangdong, Guangzhou, China.

**Keywords:** septic shock, Shen fu injection

## Abstract

**Background:**

Septic shock is a serious disease with high morbidity, which will lead to organ dysfunction. Shen fu injection (SFI) has been widely used for septic shock as a treatment in China. Many clinical trials have been reported that it could assess the efficacy and safety of SFI to cure septic shock and accelerate resuscitation. Reassessing the efficacy and safety of SFI as a treatment of patients with septic shock is the objective of this updated systematic review.

**Methods:**

The following electronic databases major in English and Chinese will be conducted a systematic search until September 2018: PubMed, EMBASE, Cochrane Library, Chinese National Knowledge Infrastructure, Chinese Science and Technology Periodical Database, Chinese BioMedical Literature Database, and Wan-fang Database. Randomized controlled trials of SFI will be included. Review Manager 5.2 software will be used for assessment of risk of bias, data synthesis, and subgroup analysis. We will conduct the GRADE system to assess the quality of evidence, if possible.

**Results:**

This study will provide a rational synthesis of current evidences for SFI on septic shock.

**Conclusion:**

We hope our research results will provide an objective and reliable evidence to patients, clinicians and healthcare policymakers who are concerning the treatment options of SFI in septic shock.

**Registration:**

PROSPERO CRD42016049332.

## 1 Introduction

Septic shock is a clinical emergency characterized by a systemic infection and perilous hypotension resulting in multiple organ dysfunction syndrome.^[1–3]^ It is reported that more than 230,000 US patients suffer from it when 40,000 US deaths happen annually.^[4]^ Conventional treatments for septic shock mainly include early administration of broad-spectrum antibiotics, multiple organ support therapy, immune regulation and inflammatory control therapy and nutritional support. However, the overall mortality of septic shock ranges from 30% to 60% despite these treatments, which has been a major burden on health services.^[5–7]^ Therefore, it is necessary to advance treatment for septic shock as well as improve its survival rate. Shen fu injection (SFI), mainly made of Red Radix Ginseng, ginsenoside, aconitine, and so on, is a well-known Chinese medicine preparation frequently used in emergency departments and intensive care units in China.^[8]^ Many studies have shown its multiple pharmacological functions of cardiac enhancement, blood pressure elevation, hemodynamic improvement, regulation of immune, inhibition of inflammatory response, and increased tolerance to hypoxia, which may lay the foundation of treating septic shock.^[9]^ Although there have been many clinical trials reporting SFI's curative effect in septic shock in China, there are still some problems in these trials such as small sample sizes and low methodological quality.^[10]^ For this reason, the objective of our study is to carry out a meta-analysis to evaluate its clinical efficacy and safety in septic shock patients and we expect that our study could provide a reference for further clinical practice.

## 2 Methods and analysis

### 2.1 Study registration

The protocol has been registered on the International Prospective Register of Systematic Reviews (PROSPERO) with registration number CRD42016049332.

### 2.2 Eligibility criteria

#### 2.2.1 Types of studies

Randomized controlled trials (RCTs) which compared SFIs with non-SFI interventions for septic shock patients, and RCTs only describe the specific random assignment method, such as random number table method will be eligible. The random assignment is mentioned, but without specific description on the method or lack clear randomization definition will not be eligible. Only rigorously controlled trials will be eligible. Non-RCTs and Quasi-RCTs such as the sequence generation according to the treatment order will be excluded.

#### 2.2.2 Types of participants

(1) Diagnostic criteria for septic shock should be followed the “sepsis campaign guidelines for management of severe sepsis and septic shock” published in 2004 (SSC 2004)^[11]^ or in 2008 (SSC 2008).^[12]^
(2) Patients with septic shock will be included regardless of their age, sex, ethnicity/race, education, or economic status.
(3) Exclusion criteria for patients will be followed.(1) Without meet diagnostic criteria of SSC 2004 or SSC 2008, (2) history of allergies for SFI, (3) active “do not resuscitate” order, (4) attending another RCTs.

#### 2.2.3 Types of interventions

Interventions will not be adopted of the control group, treating in conventional ways and routine care such as fluid resuscitation, anti-infection, nutrition support, and so on.

For the treatment group, SFI interventions will be adopted, which combine with conventional therapy, and pharmacotherapy in the treatment groups should be in accordance with control groups. Furthermore, there will not be other therapies in the treatment groups. Trials include any other Chinese medicine interventions (eg, decoction of Chinese medicine, acupuncture, etc) will be excluded.

### 2.3 Types of outcome assessments

#### 2.3.1 Primary outcomes

Mean arterial pressure (MAP), serum lactic acid, acute physiology and chronic health evaluation II (APACHE II) scores.

#### 2.3.2 Secondary outcomes

Heart rate (HR) and mortality.

### 2.4 Search methods for identification of studies

#### 2.4.1 Electronic searches

We will perform a systematic search of the following electronic databases major in Chinese and English: PubMed, Cochrane Library, EMBASE, Chinese BioMedical Literature Database, Chinese National Knowledge Infrastructure, Chinese Science and Technology Periodical Database, and Wan-fang Database from inception to September 2018.

The subject terms and keywords will be used to search the English databases: (“Septic Shock” OR “Systemic Inflammatory Response Syndrome”) and (“Shen fu injection” OR “Shen fu” OR “Shenfu”). Other English databases will apply to similar search strategies.

We will employ the Chinese search terms to search the Chinese databases: (“gan ran xing xiu ke” OR “nong du xing xiu ke” OR “quan shen yan zheng fan ying zong he zheng”) and (“shen fu zhu she ye” OR “shen fu”).

## 3 Other resources

International Clinical Trials Registry Platform and Chinese Clinical Trial Registry and the reference section of each study will also be searched. Moreover, we will obtain relevant conference papers on SFI of septic shock.

### 3.1 Data collection and analysis

#### 3.1.1 Selection of studies

All studies will be screened by 2 reviewers (YC and CF) independently. The 2 reviewers will perform the data extraction including the basic information of studies (authors, nation, year of publication, multicenter or single-center, etc), patients (baseline data, diagnostic criteria, etc), methods (registry platform, sample size, blinding, Guidelines for management, etc), interventions (types of control, dosage of SFI, treatment period of SFI, etc), and outcomes (observation index, follow-up, adverse events, etc). Disagreement between reviewers will be resolved by discussion until a consensus was reached. Third-party author (TW) will be consulted if discrepant data on retrieved article still exist. EndNote X7 software will be used to manage the literature.

#### 3.1.2 The solution to missing data or unclear scale type

**Figure 1:**
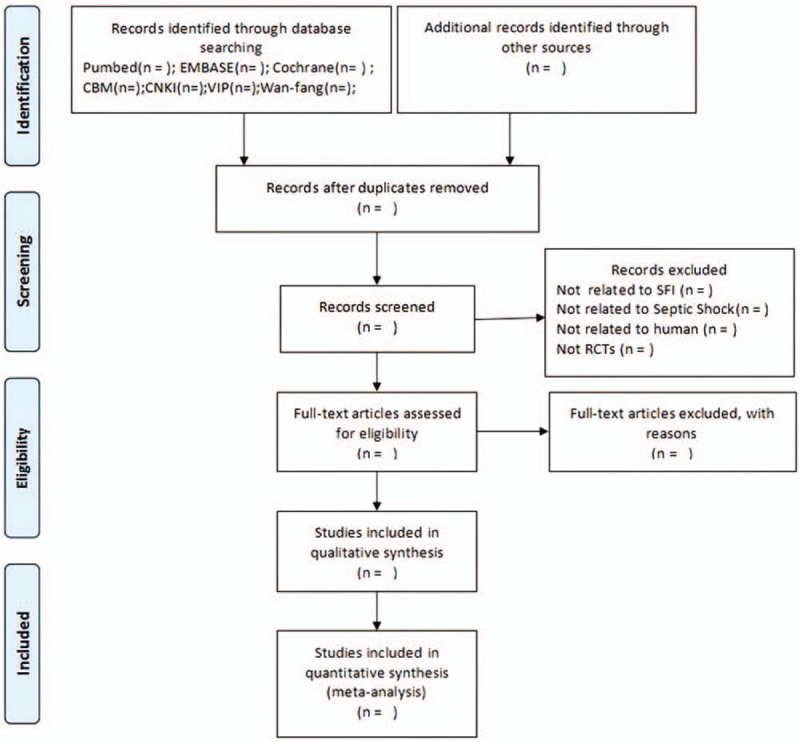
Flow diagram of selection process.

We will send an email or make a telephone call on original authors for requiring missing data or unclear evaluation scales. If this way cannot obtain the sufficient information, we will just analyze the available data. We will add the potential impact on review results which can be seen in the discussion section with insufficient data. Study selections will be shown in a PRISMA flow chart (http://www.prisma-statement.org) (Fig. 1)

#### 3.1.3 Assessment of bias in the included studies

The methodological quality of the included studies will be evaluated using the Cochrane Handbook version 5.2. At the same time, the risk of bias of all included RCTs will be assessed according to the Cochrane collaboration's tool, including 8 domains: random sequence generation; allocation concealment; the blinding of participants, personnel, and outcome assessor; incomplete outcome data; and selective reporting and other sources of bias.

#### 3.1.4 Data synthesis and statistical analysis

We will use the Cochrane Collaboration Review Manager Software (RevMan version 5.2) for meta-analysis and statistical analyses. Odds ratio and the associated 95% confidence intervals (CIs) will be calculated for mortality. The standard mean difference with 95% CIs for continuous data will be calculated, such as MAP, HR, APACHE II scores, and serum lactate. We will use a standard chi-square test and Higgins *I*^2^ statistic to estimate the results of heterogeneity between trials. The random effect models will be adopted if data with statistical heterogeneity (*P* < .1 and *I*^2^ > 50% indicate the presence of heterogeneity); otherwise, we will use a fixed-effect model.

#### 3.1.5 Subgroup analyses

We plan to conduct subgroup analyses of comparison between SFI interventions and controls. We will stratify the results by treatment duration, dosage of SFI if enough comparable data is confirmed. We will focus on different times of the outcomes, such as MAP at 1, 6, 12, 24 hour, and so on, after treatment of SFI.

### 3.2 Publication bias

The assessment of publication bias will be explored via funnel plots in RevMan V.5.2.

#### 3.2.1 Quality of evidence

We will consider the quality of evidence according to the GRADE system. Therefore, the assessment of the results will be further evaluated.

#### 3.2.2 Ethics and dissemination

This systematic review will be published in a peer-reviewed journal. Our result will provide evidence of the efficacy and safety of SFI for septic shock. This review will not related to ethics due to all data with no concerns regarding privacy.

## 4 Discussion

There is a long history that Chinese herbal medicine could treat disease since ancient times in East Asia. Now, the injections of active ingredients of traditional Chinese medicine for the clinical application has been widely used in China.^[13]^ Therefore, it is necessary to conduct the methodologies to guarantee the safety and efficacy of herbal medicines.

SFI is a preparation of Shen fu decoction, which consists of ginsenoside and aconitine.^[14]^ Pharmacological researches indicated that SFI could enhance adrenocortical system,^[15]^ dilate coronary arteries, improve coronary flow,^[16]^ enhance energy metabolism, suppress cell apoptosis,^[17]^ downregulate inflammatory cytokines, and enhance immunity.^[18]^ Furthermore, SFI plays a pivotal role in regulating the post-resuscitation immune dysfunction,^[19]^ promoting the biosynthesis of proteins,^[20–22]^ as well as scavenging free radicals,^[23]^ reducing calcium overload, improving heart contractility,^[24]^ attenuating reperfusion injury by alleviating swelling of mitochondria and cells.^[25]^

Study also shows that the cardioprotective effect of SFI may be in part related to increasing superoxide dismutase activity and improving the pathologic changes of myocardium.^[26]^

According to a number of studies, a review published recently^[27,28]^ evaluate the safety and efficacy of SFI for septic shock during the past decade. However, most of the included studies were in a low methodological quality (especially without clear randomization definition) and all of the literature was written in Chinese language. Moreover, some rigorously controlled trials and new data of SFI applied for septic shock have been published. Thus, it is significant to restart a comprehensive systematic review to assess the efficacy and safety of SFI for patients who are suffering from septic shock. In this review, the efficacy and safety of SFI for Septic Shock will be reassessed. We hope our research results will provide an objective and reliable evidence to patients, clinicians and healthcare policymakers who are concerning the treatment options of SFI in septic shock.

## Author contributions

**Conceptualization:** Xiaojun Wang.

**Formal analysis:** Canfeng He.

**Methodology:** Yinhe Cai.

**Resources:** Dazhong Sun.

**Software:** Guangyun Hu.

**Writing – original draft:** Xiaojun Wang, Guangyun Hu.

**Writing – review & editing:** Jian Zhou.

Xiaojun Wang orcid: 0000-0002-7613-692X.
